# Biomarkers of Ovarian Reserve

**DOI:** 10.4137/bmi.s537

**Published:** 2008-04-16

**Authors:** William E. Roudebush, Wendy J. Kivens, Jessica M. Mattke

**Affiliations:** Beckman Coulter, Inc., Immunoassay Business Group, Chaska, MN, U.S.A

**Keywords:** ovarian reserve, FSH, inhibin-B, AMH/MIS

## Abstract

The primary function of the female ovary is the production of a mature and viable oocyte capable of fertilization and subsequent embryo development and implantation. At birth, the ovary contains a finite number of oocytes available for folliculogenesis. This finite number of available oocytes is termed “the ovarian reserve”. The determination of ovarian reserve is important in the assessment and treatment of infertility. As the ovary ages, the ovarian reserve will decline. Infertility affects approximately 15%–20% of reproductive aged couples. The most commonly used biomarker assay to assess ovarian reserve is the measurement of follicle stimulating hormone (FSH) on day 3 of the menstrual cycle. However, anti-müllerian hormone and inhibin-B are other biomarkers of ovarian reserve that are gaining in popularity since they provide direct determination of ovarian status, whereas day 3 FSH is an indirect measurement. This review examines the physical tools and the hormone biomarkers used to evaluate ovarian reserve.

## Introduction

The primary function of the female ovary is the production of a mature and viable oocyte capable of fertilization and subsequent embryo development and implantation. At birth, the ovary contains a finite number of oocytes available for folliculogenesis. This finite number of available oocytes is termed “the ovarian reserve”. The determination of ovarian reserve is important in the assessment and treatment of infertility. As the ovary ages, the ovarian reserve will decline.

Ovarian reserve (OR) refers to the number and quality of oocytes that, at any given age, are available to produce a dominant follicle late in the follicular phase of the menstrual cycle. By estimating the OR, a prediction of the remaining reproductive lifetime could be assessed as well as the likely success of assisted reproductive techniques (ART) such as *in vitro* fertilization (IVF) (Baird et al. 2005). None of the OR tests directly measures the total number of actual oocytes. Rather, it is assumed that the number of recruitable and developing follicles (pre-antral and antral) is directly related to the total oocyte pool. During fetal life the ovaries are endowed with the entire stock of follicles (oocytes surrounded by ovarian granulosa cells) that will serve a woman’s reproductive life. Because the number of quality oocytes available for recruitment during folliculogenesis changes markedly during a woman’s lifetime, the tendency is for OR physical evaluation tests to inaccurately estimate the total pool of “good-viable” oocytes. This review will look at the physical tools utilized to determine ovarian reserve.

Infertility affects approximately 15%–20% of reproductive aged couples. The most commonly used biomarker test to assess ovarian reserve is the measurement of day 3 follicle stimulating hormone (FSH); this blood test determines the level of FSH on day 3 of the menstrual cycle. Cycle day 3 is the preferred testing day due to the expected low level of estradiol, which in turn affects FSH levels via negative feedback control. Therefore, Day-3 FSH levels would be expected to be low, a higher than normal day-3 FSH level would indicate a diminished ovarian reserve. However, this day still requires standardization to ensure reproducibility. Typically, Day-3 FSH and estradiol are both measured. However, other blood tests (antimüllerian hormone and, or inhibin-B) are gaining popularity since they provide more direct determination of ovarian status, whereas Day-3 FSH and estradiol are indirect measurements. This review will look at the aforementioned hormones as biomarkers of ovarian reserve.

## Overview of Reproductive Endocrinology

From the hypothalamus, gonadotropin-releasing hormone (GnRH) acts upon the anterior pituitary to produce both FSH and luteinizing hormone (LH) both of which target the ovary in females. FSH is responsible for follicular recruitment and growth and for estrogen (mostly β-estradiol) conversion from androgens during folliculogenesis. Estrogens (e.g. estradiol, E_2_) are the primary hormones that provide negative feedback to the hypothalamus and anterior pituitary to inhibit FSH and LH secretion. Granulosa cells are the target cells within the ovary for FSH activity. LH is responsible for final follicular and oocyte maturation, subsequent ovulation, and corpus luteum (CL) formation. During folliculogenesis, LH acts upon the ovarian theca cells to produce androgens. Following ovulation, LH will promote estrogen and progesterone secretion by the CL. In addition to steroidal hormones, the ovary (i.e. granulosa cells) also produces a number of peptide hormones of the transforming growth factor (TGF)-β/activin superfamily. These peptides hormones may include relaxin, inhibin A, inhibin B, activin, follistatin and antimüllerian hormone (AMH) also called Müllerian inhibiting substance (MIS). For the purpose of this review, we will refer this peptide hormone as AMH.

The actions of activin include granulosa cell proliferation, upregulation of FSH and LH receptor expression, enhancement of aromatase activity and subsequent estradiol production, suppression of androgen production, increased production of inhibins and follistatin and the enhancement of oocyte developmental competence. The primary action of follistatin is to bind to and neutralize activin.

Inhibin consists of two distinct chains, or subunits (alpha and beta), linked together. Inhibin A consists of the alpha-subunit and beta A-subunit. Inhibin B consists of the alpha-subunit and beta B-subunit. Only the dimeric forms of the molecule, containing both the alpha and beta subunits, are bioactive, although the free subunit forms exist in circulation. Inhibins are secreted by ovarian granulosa cells in females and by testicular Sertoli cells in males. Both inhibin A and inhibin B are produced in females, but in males inhibin B is the major circulating inhibin. Both inhibins suppress FSH secretion from the pituitary. During the menstrual cycle and early pregnancy, inhibin A is produced by the CL. At the onset of menstruation during the early follicular phase, very low levels of inhibin A are detected. Levels of inhibin A increase dramatically in the late follicular phase and peaks in mid-luteal phase. The primary role of inhibin B appears to be in the regulation of folliculogenesis via a negative feedback on the production of FSH. Levels of inhibin B increase dramatically during folliculogenesis and are maximized just prior to ovulation.

AMH is produced by the Sertoli cells of the testis in the male and exclusively by ovarian granulosa cells of preantral follicles in the adult female. During embryonic development in males, secretion of AMH from testicular Sertoli cells is essential for the regression of the Müllerian ducts, and thus the normal development of the male reproductive tract. The Müllerian ducts are the primordium for the uterus, Fallopian tubes and upper vagina in the female. In the male, secretion of AMH by the Sertoli cells commences during embryogenesis and continues throughout life. Levels drop following puberty, decreasing slowly to a relatively low post-puberty value. In the female, serum AMH is undetectable until the onset of puberty. AMH is produced in a wide range of follicles from primary to early antral stages of folliculogenesis. The role of AMH is to modulate primordial follicle recruitment and to inhibit cyclic follicle recruitment for folliculogenesis, primarily by inhibiting the action of FSH on follicle growth and selection. AMH levels are maintained at relatively low levels until menopause, at which time AMH progressively decreases again to undetectable levels.

## Ovarian Reserve and Physical Testing

### Ovarian reserve (OR) declines during the aging process

Although a decline in OR accompanies chronological aging, acceleration in this process appears to occur in a subset of pre-menopausal women. This is evidenced by suboptimal responses to ovarian stimulation attempts, as seen in a proportion of younger women undergoing treatments for infertility (de Boer et al. 2002; de Boer et al. 2003) and by the recent recognition that this subset of women may be destined for transition into menopause at a younger age (Lawson et al. 2003).

The determination of both the quantity and quality of the follicular pool may allow the prediction of women who may under-respond or over-respond to controlled ovarian hyperstimulation protocols in ART programs (Bukulmez and Arici, 2004; Chang et al. 1998; Frattarelli et al. 2003; Bancsi et al. 2002; Vladimirov et al. 2005).

### Physical tools used to assess OR

Ultrasonography (US) may be a useful tool in evaluating current ovarian function. During the early follicular phase of the menstrual cycle, the measurement of the ovarian volume (Lass et al. 1997), the mean ovarian diameter (MOD) (Frattarelli et al. 2000), the antral follicle count (AFC) (Ruess et al. 1996), and the ovarian stromal blood flow with color Doppler (Engmann et al. 1999; Zaidi et al. 1996) are all physical evaluation techniques for ovarian reserve prediction.

Ovarian volume and AFC values can be useful indicators of menopausal status (Flaws et al. 2001). Erdem et al. suggest that transvaginal ultrasonography (TVS) rather than hormonal parameters is the preferred method for OR determination, as TVS assessment of ovarian volume and the AFC confer a stronger correlation with chronological aging than Day 3 FSH level indices and aging (Erdem et al. 2003).

The use of sonographic methods are somewhat limited, however, as they cannot predict future fertility. Rather, US can only predict current fertility or the ovarian response to IVF treatment. Until the very late stages of reproductive aging, most sonographic cycle characteristics in populations with proven fertility remain “normal” (te Velde and Pearson, 2002). Only at a mean age of approximately 46 to 48 years do normal menstrual cycle characteristics tend to disappear (te Velde and Pearson, 2002; Giacobbe et al. 2004).

In younger women, sonographic methods may be only a fertility snapshot during one menstrual cycle, as cycle-to-cycle consistency of both the AFC and ovarian volume have been demonstrated to be significantly variable in younger sub-fertile women, with more variation observed in the AFC of younger infertile patients (Elter et al. 2005). Hence, a low AFC in a young, sub-fertile ovulatory woman should be interpreted cautiously, as a low AFC may not reflect poor ovarian reserve.

### Ovarian volume measurements

The ovarian volume in terms of total ovarian volume, volume of the smallest ovary and mean ovarian volume, measured by TVS, were reported to correlate with response to controlled ovarian hyperstimulation (Lass et al. 1997; Flaws et al. 2001; Sharara and McClamrock, 1999; Syrop et al. 1999). In women where either ovary is small (less than three cm^3^), the IVF cancellation rate was higher (Sharara and McClamrock, 1999). Single ovarian dimensions were shown to be a reliable predictor of declining OR status in pre-menopausal infertile women (Bowen et al. 2007) and in ovarian responsiveness during ART cycles (Frattarelli et al. 2000; Frattarelli et al. 2002). The magnitude of this association was most robust for the ovarian width measurement (Bowen et al. 2007).

Some studies detected a significant negative correlation between age of infertile women and ovarian volume by two-dimensional US (Syrop et al. 1999) and by three-dimensional US (Kupesic et al. 2003); other studies could not demonstrate such a correlation (Sharara and McClamrock, 1999; Syrop et al. 1995). In fact, in a family planning clinic population of healthy women aged 14–45 years, ovarian volume was shown not to be related to age (Christensen et al. 1997). Additional studies have demonstrated that a progressive decrease in ovarian volume correlated to aging is more discernable during post-menopause and not during the reproductive age, suggesting that ovarian volume should not be utilized as a stand-alone OR test (Tepper et al. 1995; Ng, 2003 #80).

### Antral follicle count measurements

The antral follicle count (AFC) is defined as the number of follicles smaller than 10 mm in diameter detected by TVS in the early follicular phase. The AFC has been shown to be a predictor of the number of oocytes retrieved in controlled ovarian hyperstimulation protocols (Tomas et al. 1997), the cancellation rates in IVF (Frattarelli et al. 2000; Tomas et al. 1997), and for predicting pregnancy loss in IVF pregnancies (Elter et al. 2005). The AFC has also been show to be a predictor of the number of immature oocytes retrieved for *in vitro* maturation (IVM) (Tan et al. 2002). There is no significant difference between right-sided and left-sided antral follicle counts within the same individual (Chow et al. 2004). The AFC was shown to be a superior, or at least an equivalent, prediction tool for poor IVF response (Hendriks et al. 2007; Kwee et al. 2007) or hyper IVF response (Kwee et al. 2007) when compared to ovarian volume measurement and complex endocrine challenge tests. Furthermore, the AFC has been established to be equally as useful as AMH in OR status determination and/or ovarian responsiveness (Muttukrishna et al. 2005; Nardo et al. 2007).

### Reproducibility/reliability of the AFC measurement

The relationship between reproductive age and AFC and the reproducibility of AFC in regularly cycling women has been investigated by a number of different groups. Healthy female volunteers with proven, normal fertility and regular menstrual cycles were studied; and out of all parameters tested, the number of antral follicles has the closest association with chronological age (Scheffer et al. 2003). Chinese women with proven fertility were evaluated; and among many ovarian reserve tests, only the AFC demonstrated the best correlation with women’s age (Ng et al. 2003).

Women undergoing their first IVF cycle were evaluated with a battery of tests to compare several basal ovarian reserve markers (Bancsi et al. 2002). Measurements were performed to determine the number of antral follicles, total ovarian volume, basal FSH, E_2_, and Inhibin B on cycle day 3. The AFC was the best single predictor for poor ovarian response (Bancsi et al. 2002). Further work by the same group demonstrated that a single AFC is a good predictor of poor ovarian response in IVF, and that the clinical relevance of a second AFC during a subsequent cycle is of limited value (Bancsi et al. 2004).

In another study, the AFC was obtained in regularly cycling fertile women and was evaluated by cohort comparison for predicted distribution of age at reproductive events, such as the ages at last childbirth and at menopause (Broekmans et al. 2004). Distribution of the observed ages at last childbirth and ages at menopause were obtained from the BALSAC (Bouchard et al. 1989) demographic database and the Prospect-EPIC (Riboli et al. 2002) study, respectively. This data comparison demonstrated that the link between declining AFCs and reproductively significant events like loss of natural fertility and menopause is strengthened by the high degree of similarity among the predicted and observed age distributions (Broekmans et al. 2004). The study authors do point out, however, that there may be marginal clinical utility, except in the case of women who have low AFCs for their age.

In a new and rather intriguing study, a number of ovarian reserve tests were performed, such as AFC and an endocrine test panel, on sub-fertile, ovulatory patients (Haadsma et al. 2007). The study demonstrated that the number of pre-antral or small antral follicles (2–6 mm) declined with age and the number of larger follicles (7–10 mm) remained constant, suggesting that the number of small AFCs represents the functional OR (Haadsma et al. 2007).

The performance of the AFC for predicting failure to achieve pregnancy is poor (Bancsi et al. 2002; Ng et al. 2003; Hendriks et al. 2005). While the AFC determines the number of oocytes, a clinically relevant outcome, such a pregnancy or live birth, is dependent on oocyte quality as well as quantity.

The ovarian volume, AFC, vascularization index, flow index, and vascularization-flow index were determined by 3-D Power Doppler Angiography (PDA) indices, and all were shown to have excellent intra-observer and inter-observer reproducibility (Merce et al. 2005). Further, the ovary functional stage (basal after pituitary suppression or stimulated after gonadotropin treatment) does not modify the reliability of any of these measurements (Merce et al. 2005). Thus, 3-D US and PDA offer the advantage of evaluating all parameters in a single US examination, thereby improving the clinical evaluation of ovarian parameters. In addition, ovarian images captured by 3-D sonography can be stored and evaluated later, thereby uncoupling the need to analyze the data during ovarian examination (Scheffer et al. 2002).

### Biomarkers: endocrine testing

Biomarkers are desirable for assessing fertility because of the minimal invasiveness of blood collection in comparison to other procedures, reflection of internal function, speed of analysis and reasonable cost. The “magic bullet” biomarker for ovarian reserve has yet to be clearly defined, yet. However many biomarkers do provide significant insight as to ovarian reserve status. With the implementation of better testing methods and discovery of new biomarkers more options will be available. There are a few newly recognized biomarkers that look extremely promising, including AMH and inhibin-B.

There are both indirect and direct measures of ovarian reserve. Indirect measures include day 3 follicular stimulating hormone (FSH), estradiol, and the FSH:LH ratios. These biomarkers are considered indirect measures, as they require stimulation from either a feedback inhibition or a stimulation loop. Essentially these biomarkers rely on the production of other hormones. Inhibin B and AMH are examples of direct measures of ovarian reserve as these hormones are produced during specific stages of follicular development, rather than by follicular stimulation. These two biomarkers have recently been receiving additional visibility with the development of more robust and reliable laboratory methods. This makes these biomarker assays additionally appealing and further reproducible for laboratories. In addition to indirect and direct measures, there are a couple of stimulation tests that have been developed and are frequently used to ascertain or estimate ovarian reserve. These stimulation tests require drug exposure, baseline measurements, and follow-up measurements of the biomarkers, such as FSH.

## Indirect Measures

### Day 3 FSH

Day 3 FSH is believed to represent the “basal” level or non-suppressed level of FSH through ovarian feedback to the pituitary (Barnhart and Osheroff, 1998). Day 3 FSH has been credited with being a biomarker for ovarian reserve since the late 1980s, as it provides a glimpse of how well the hypothalamic-pituitary-gonadal axis is functioning (Barnhart and Osheroff, 1998). As women and their follicles age, the amount of FSH secreted increases due to the lack of responsiveness of the ovary (Perloe et al. 2000). As day 3 FSH levels climb, it is indicative of a diminished ovarian reserve. In fact, fluctuation between cycles of day 3 FSH levels is important to note as it may be reflective of the decline in ovarian reserve (Perloe et al. 2000).

Monitoring day 3 FSH levels may not be the best option for monitoring ovarian reserve, but it is the most widely recognized OR biomarker and it does provide some insight. Testing is available on multiple automated platforms and thus is relatively fast, inexpensive, and reproducible. FSH is proven to increase with the age of follicles and that increase is more dramatic and earlier than that of LH (Perloe et al. 2000). Historically, FSH has been the biomarker of choice; it is well studied, documented, and validated which provides a level of comfort to physicians (Sharara et al. 1998).

It is important to recognize some of the issues with using FSH as a biomarker for OR testing. Between cycle fluctuation in day 3 FSH levels make OR estimation difficult (Perloe et al. 2000). Since lower day 3 FSH levels represent satisfactory ovarian reserve and higher levels represent declining OR, a single day 3 FSH measurement may not be very accurate. It may be better to look at subsequent cycle day 3 FSH levels (Perloe et al. 2000). Additionally, an increased day 3 FSH level is considered a late indicator of marked decreased fertility potential (Sharara et al. 1998). It may be better to look for an early indicator of declining OR and/or decreased fertility potential.

### Estradiol

Day 3 estradiol has been assessed for OR testing as well, but is not as extensively relied upon. Estradiol is a product of the granulosa cells and can be considered a reflection of follicular activity. As with FSH, estradiol testing is also available on multiple automated platforms and thus is relatively fast, inexpensive and reproducible. However, it is never used alone as a biomarker for OR.

An increased estradiol level early in the menstrual cycle suggests that follicular development is in an advanced stage that is inappropriate for day 3 (Perloe et al. 2000). However, estradiol levels can be increased for two very different reasons. Estradiol levels can become elevated due to the occurrence of rapid folliculogenesis. Alternatively, an increased estradiol level can be due to an enhanced OR, such as in women afflicted with polycystic ovarian syndrome (PCOS), where a small amount of estradiol is being produced by a large number of antral follicles (Toner, 2003).

### FSH:LH ratio

The literature gives some honorable mention to looking at the ratio of measured FSH to LH, which is most frequently determined on day 3 of the cycle. An elevated or exaggerated FSH:LH ratio can be a signal of diminished OR (Toner, 2003). By looking at the ratio an elevation can be detected, even with an FSH level that appears to be within the reference interval (Toner, 2003). Since FSH begins rising before LH as OR diminishes, using two measurements may be more reliable and will catch a small increase in FSH faster (Perloe et al. 2000). The FSH:LH ratio is an early indicator of ovarian ageing and could be the first of diminished OR (Mukherjee et al. 1996).

## Direct Measures

### Inhibin B

Inhibin B is a peptide hormone that is a member of the transforming growth factor-β (TGF-β) superfamily (Perloe et al. 2000). It is produced from small antral follicles and selectively inhibits FSH release (Perloe et al. 2000; Broekmans et al. 1998). Inhibin B levels during the early follicular phase decrease prior to the increase in FSH levels (Toner, 2003). As the follicle cohort size is decreased, which is expected as women age, a decrease in inhibin B levels will be observed (Broekmans et al. 1998).

Unfortunately, documentation in the literature is minimal and also variable. Inhibin B levels do hold promise, but need more study and validation (Sharara et al. 1998). This could be attributed to a variation between assays used and there may be concerns about cycle-to-cycle variability (Sharara et al. 1998). One study of interest found inhibin B to have the best positive association with the number of oocytes collected from patients undergoing gonadotropin ovarian stimulation tests to assess ovarian reserve (Muttukrishna et al. 2005). This study looked at 108 women and the change in inhibin B between days 3 and 4, as well as other biomarkers. Currently, there is only one commercially available assay for inhibin B. Unfortunately, it is still being optimized and is currently available for research use only.

### AMH

Like inhibin B, antimüllerian hormone (AMH) is also a member of the TGF-β superfamily. AMH is produced by the granulosa cells of pre-antral and small antral follicles. Follicular growth is modulated by AMH, which inhibits recruitment of follicles from the primordial pool by modifying the FSH sensitivity of those follicles (La Marca et al. 2006; Visser et al. 2006). AMH is considered to be reflective of the non-FSH dependant follicular growth. As a follicle matures, AMH production disappears allowing the follicle to complete the development process during the FSH-dependant stages of growth (Visser et al. 2006). There is a linear decline of AMH levels over time (Visser et al. 2006; La Marca and Volpe, 2006). This decline is attributed to a decreasing number of follicles in the primordial pool.

AMH will in all probability become the hormone of choice for assessing OR. It has been suggested that AMH is the single best predictor of poor response for ART (Muttukrishna et al. 2005). The fact that AMH is secreted without dependence on other hormones, particularly the gonadotropins, and that AMH is expressed at a constant level, independent of cycle day make AMH very attractive as a direct measurement of OR (La Marca et al. 2006; Feyereisen et al. 2006; Hehenkamp et al. 2006; La Marca et al. 2007). The freedom that AMH testing offers both clinicians and patients by allowing collections to be performed on any day during the menstrual cycle is a vast logistical advantage over other biomarkers.

One recent study demonstrated not only a strong relationship between AMH and AFC, but that this relationship was stronger than the other typical biomarkers relationships with AFC (Feyereisen et al. 2006). In addition to being a good biomarker for the quantity of follicles, another study illustrated that AMH is also suggestive of the quality of the remaining oocytes (Ebner et al. 2006). Women with normal reproductive performance were examined twice within an average of four years and assessed the AFC and various endocrine markers, demonstrating that serum AMH, followed by the AFC showed the most consistent correlation to the age-related decline of reproductive capacity (van Rooij et al. 2005).

Additional research is needed to recognize all the roles AMH plays. It will be important to recognize the mechanisms that control production of AMH within the granulosa cells (Feyereisen et al. 2006). To better understand if AMH is truly reflective of quality of the follicles, the fate of oocytes and embryos from individual follicles will need to be assessed (Feyereisen et al. 2006).

### Clomiphene citrate challenge test

It is thought by some that provocative or stimulation tests for ovarian reserve testing are more sensitive indicators than basal testing alone (Toner, 2003). The Clomiphene Citrate Challenge Test (CCCT) is perhaps the most widely used of the stimulation tests for ovarian reserve. The underlying assumption for this test to work is that in a woman with adequate ovarian reserve, there is a healthy developing follicle cohort that should be able to produce enough inhibin and estradiol to suppress FSH and resist the clomiphene citrate stress on the hypothalamus-pituitary-gonadal axis (Perloe et al. 2000).

The CCCT requires a baseline measurement of FSH on day 3 of the cycle, administration of 100 mg of clomiphene citrate daily on cycle days 5 through 9 and an additional measurement of FSH on cycle day 10. If the FSH determination on day 10 is high, this result is suggestive of a diminished OR. In fact, some users of the CCCT look at both day 3 and day 10 FSH levels independently and others additively. If either or both measurements are high, these results are also indicative of a diminished OR (Perloe et al. 2000; Toner, 2003; Hendriks et al. 2005). When applied in an ART setting, the CCCT has been proven to be a better predictor of OR than day 3 FSH measurement alone (Perloe et al. 2000).

There is additional burden placed upon the patient when undergoing a CCCT. The two visits and administration of the drug for 5 days require patient compliance that can affect the accuracy of the CCCT (Hendriks et al. 2005). These patients do have a vested interest, so compliance may be higher than in other situations; however it is still an unknown variable that must be considered in evaluating the use of the test. In addition, there may be no need to perform the CCCT if there is an elevated day 3 FSH measured (Broekmans et al. 1998).

### GAST

The Gonadotropin-releasing Hormone Agonist Test (GAST) is another stimulation test that is fairly well documented in the literature. It evaluates the change in estradiol levels on cycle days 2 and 3 following a subcutaneous administration of a gonadotropin-releasing hormone agonist (1 mg leuprolide acetate) (Perloe et al. 2000). The dose of the agonist causes a massive, temporary release of FSH and LH from the pituitary, which in turn increases estradiol production within a 24-hour timeframe (Broekmans et al. 1998). A robust increase or flare of estradiol in response to this stimulation is reflective of recruitable follicles in the early follicular phase which is in turn representative of OR (Perloe et al. 2000; Broekmans et al. 1998).

## Summary

- All tests have some benefits. There will always be a combination of markers used to get the best answer. The best candidate for a single biomarker is AMH.
- We could recommend the need of a reference interval study using a “normal” population in addition to an infertile population. Reference intervals for each population should be stratified by age and body mass index and for ART patients, further stratified by gonadotropin response, i.e. poor versus good responder.
- Bottom line: AMH is the focus for the future of ovarian reserve assessment. Further clinical studies are needed, but today, AMH appears to be representative of the hands of the biological clock that we have been hearing tick for years.

## Figures and Tables

**Figure 1:**
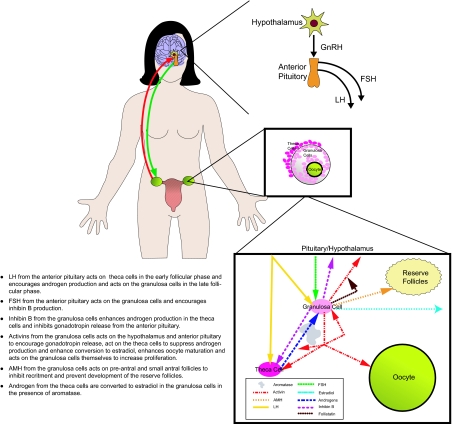
Schematic of the reproductive endocrinology in the female. Please see notations within the Figure for detailed description of the relationships between the hormones.

**Table 1:** Comparison of the different physical tools to assess ovarian reserve.

Physical measurement tool	Advantages	Disadvantages
Ovarian volume	Confirms menopausal status reliable predictor of declining OR status independent of advancing age	Significant changes in ovarian volume not discernable during end of reproductive years Highly variable in younger infertile patients
Antral follicle count	Consistent correlation to the age-related decline of reproductive capacity There is no significant difference between right-sided and left-sided antral follicle counts within the same individual The AFC is a useful prediction tool for poor IVF response or hyper IVF response	Performance for predicting failure to achieve pregnancy is poor Highly variable in younger infertile patients AMH may be a better/equivalent predictor of age-related decline of reproductive capacity
Ovarian stromal blood flow velocity	Results are highly predictive of ovarian responsiveness	Results do not always correlate with advancing age
